# Supplementary material for: Salivary Proteomics Markers for Preclinical Sjögren’s Syndrome: A Pilot Study
Source: Biomolecules. 2022 May 24;12(6):738. doi: 10.3390/biom12060738 (PMC9221050; doi:10.3390/biom12060738)

Supplementary Materials

Title: Salivary Proteomics Markers for Preclinical Sjögren's Syndrome: A Pilot Study

Nicoletta Di Giorgi[1,&,*], Antonella Cecchettini[1,2,&], Elena Michelucci[1], Giovanni Signore[3,4], Elisa Ceccherini[1], Francesco Ferro[2], Elena Elefante[2], Chiara Tani[2], Chiara Baldini[2,†,*] and Silvia Rocchiccioli[1,†]

[1] Clinical Phisiology Institute-CNR, 56124, Pisa, Italy; digiorgi@ifc.cnr.it (N.D.G.); antonella.cecchettini@unipi.it (A.C.); emichelucci@ifc.cnr.it (E.M.); silvia.rocchiccioli@ifc.cnr.it (S.R.)
[2] Department of Clinical and Experimental Medicine, Rheumatology Unit, University of Pisa, 56126, Pisa, Italy; francescoferrodoc@gmail.com (F.F.); elena.elefante87@gmail.com (E.E.); chiaratani78@gmail.com (C.T.); chiara.baldini@unipi.it (C.B.)
[3] Department of Biology, Biochemistry Unit, University of Pisa, 56126, Pisa, Italy; giovanni.signore@unipi.it (G.S.)
[4] Fondazione Pisana per la Scienza Onlus, via Ferruccio Giovannini 13, 56017 San Giuliano Terme, Italy; g.signore@fpscience.it (G.S.)
[&] These two authors equally contributed to the study.
† These two authors are co-last authors.
* Correspondence: digiorgi@ifc.cnr.it (N.D.G.); chiara.baldini@unipi.it (C.B.)

Table of contents

Figure S1: Size distribution by intensity of EV-enriched saliva samples. 3

Table S1: 40 Q1 variable windows covering 250 - 1250 m/z range. 4

Table S2: Selected endogenous peptides from top score proteins used for the retention time alignment of library and SWATH-MS samples..5

Figure S2: GO enrichment analysis results for biological processes (BPs). 6

Figure S3: GO enrichment analysis results for molecular functions (MFs)..7

Figure S4: GO enrichment analysis results for cellular components (CCs). 7

Figure S1: Size distribution by intensity of EV-enriched saliva samples. Size distribution and relative percentage abundance (determined based on intensity distribution) of saliva-enriched samples used for the analysis. Reported size distribution is the average of three experiments. Errors are calculated based on the PDI of each vesicle population.

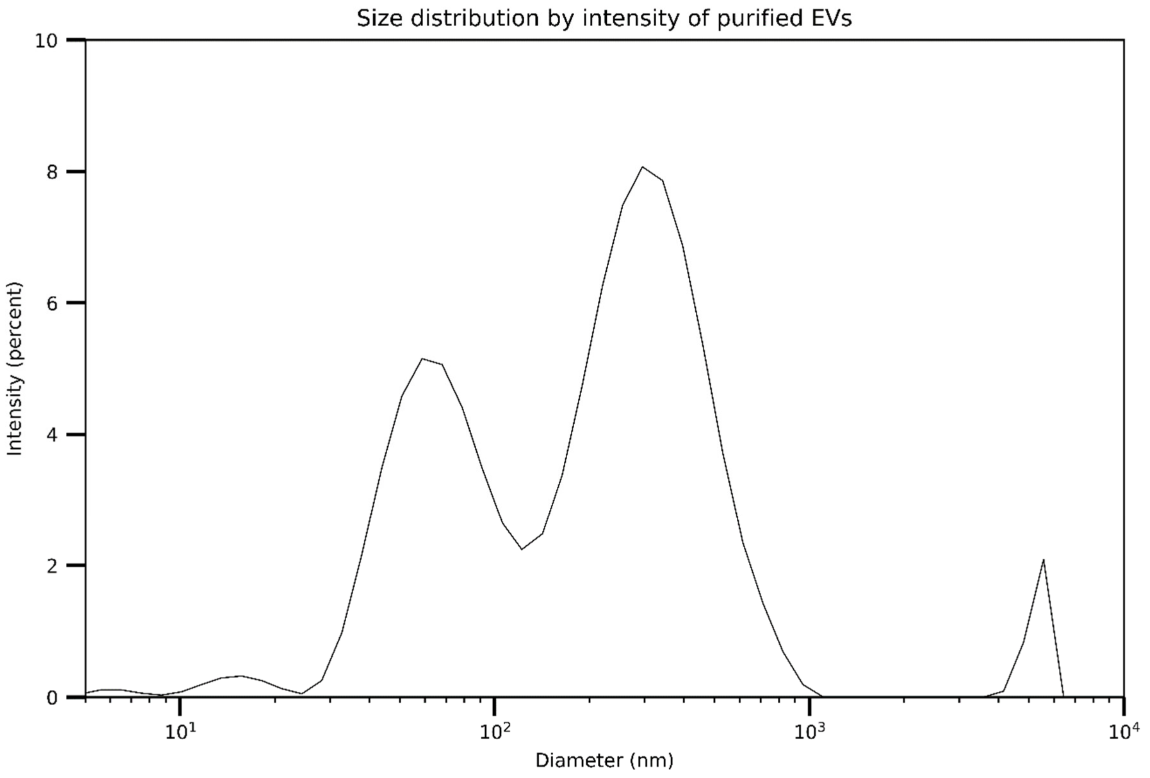

Table S1: 40 Q1 variable windows covering 250 - 1250 m/z range.

Start	End
249.5	269.5
268.5	286.5
285.5	303.5
302.5	320.5
319.5	336
335	349.5
348.5	362
361	374
373	385
384	396
395	406
405	416
415	426
425	435.5
434.5	445
444	454
453	463
462	472
471	481.5
480.5	491.5
490.5	502
501	513.5
512.5	526
525	539.5
538.5	554
553	568.5
567.5	583
582	597.5
596.5	612
611	627.5
626.5	645
644	666.5
665.5	693.5
692.5	721
720	748.5
747.5	781.5
780.5	827.5
826.5	896
895	1008.5
1007.5	1249.5

Table S2: Selected endogenous peptides from top score proteins used for the retention time alignment of library and SWATH-MS samples.

Peptides
EVTINPDTTC[CAM]GNDWVC[CAM]EHR
SVTWSESGQGVTAR
TFTC[CAM]TAAYPESK
LTPLQFGNLQK
LSPSC[CAM]PDALAPK
AFVNC[CAM]DENSR
LTDPNSAFSR
TVTINC[CAM]PFK
LSC[CAM]LGASLQK
AQAQPGVPLR
IIEGEPNLK
TWLVPDSR
GVQLSDWR
TFDGDVFR
SGNEDEFR
IYVSDDGK
TAAYPESK

Figure S2: GO enrichment analysis results for biological processes (BPs). GO enrichment analysis results for BPs showed significant enrichment of process involved in the activation of immune system, particularly of humoral immunity, with activation of B lymphocytes and secretion pathways, and innate immunity, with activation of inflammatory processes. (**a**) Enriched BPs were provided in output as a graph, where each node represented a biological process and the colour intensity of nodes increased with statistical significance. In addition, BPs were clustered according to common proteins to improve the understanding of results. Each cluster contains related BPs. (**b**) Most statistically significant enriched BPs are reported in a bar plot with their relative significance (-log10p-value on X-axis) and their relative number of proteins in the input data (Proteins as colour scale).

(a)

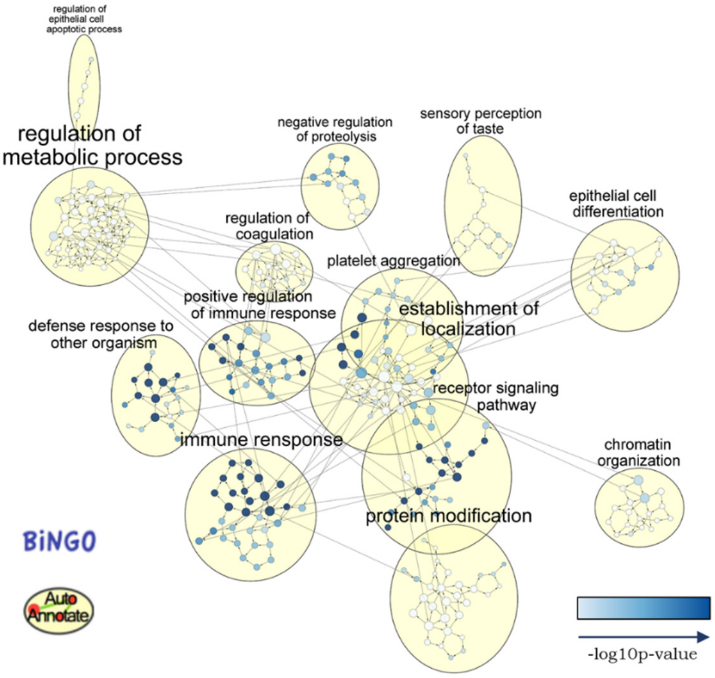

(b)

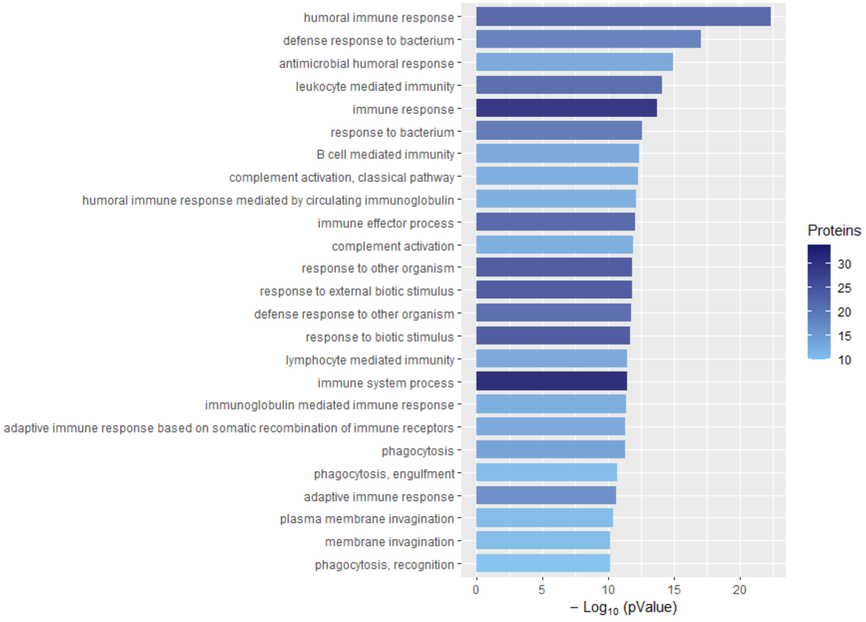

Figure S3: GO enrichment analysis results for molecular functions (MFs). GO enrichment analysis results for MFs showed that most statistically significant enriched MFs were antigene and immunoglobuline receptor binding, together with endopeptidase regulator activity. Results from this analysis are reported in a bar plot with their relative significance (-log10p-value on X-axis) and their relative number of proteins in the input data (Proteins as colour scale).

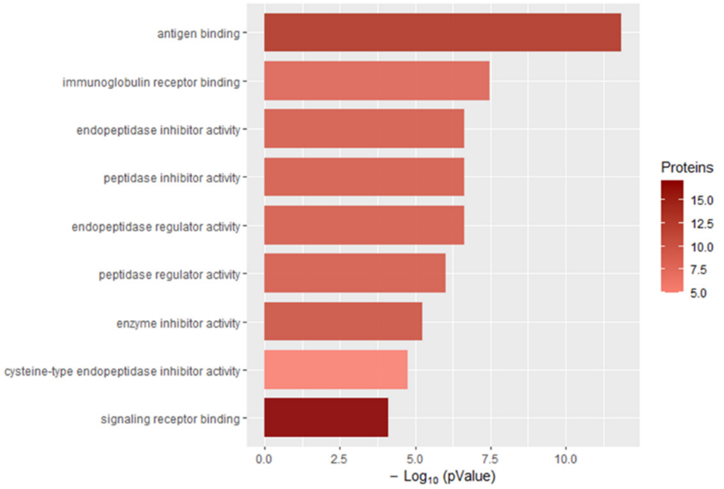

Figure S4: GO enrichment analysis results for cellular components (CCs). From GO enrichment analysis on CCs emerged as most statistically significant enriched extracellular regions, with about half of the differential expressed proteins attributable to extracellular vescicles and a high significance resulted also for immunoglobuline complexes. Statistically significant enriched CCs are reported in a bar plot with their relative significance (-log10p-value on X-axis) and their relative number of proteins in the input data (Proteins as colour scale).

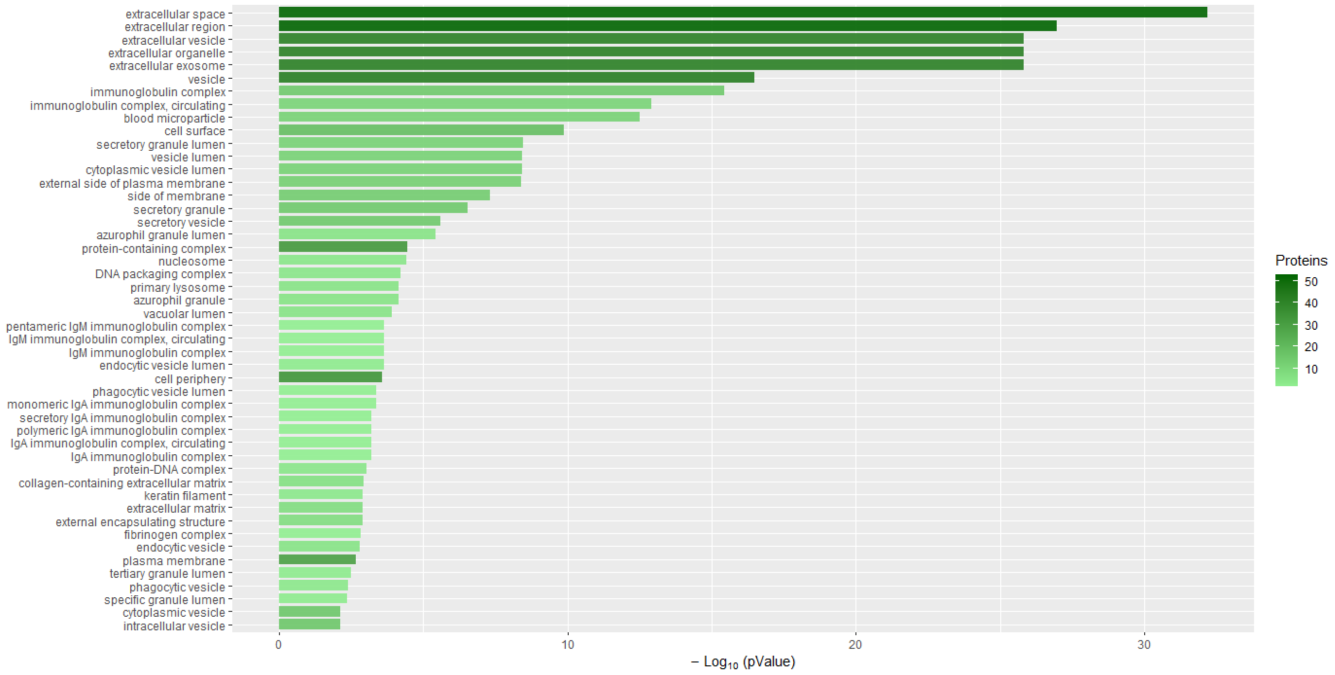

Supplement: Supplementary file 1 [file biomolecules-12-00738-s001.zip › SupplementaryMaterials.pdf]
